# Knee Sepsis after Suprapatellar Nailing of an Open Tibia Fracture: Treatment with Acute Deformity and External Fixation

**DOI:** 10.1155/2019/3185286

**Published:** 2019-01-06

**Authors:** Chelsea E. Minoughan, Adam P. Schumaier, Frank R. Avilucea

**Affiliations:** ^1^University of Cincinnati Department of Orthopaedics and Sports Medicine, Cincinnati, OH, USA; ^2^Orlando Health Orthopedic Institute Orthopedic and Sports Medicine Group, Orlando, FL, USA

## Abstract

**Case:**

A 31-year-old male was involved in a dirt bike accident and sustained an isolated type II open mid-distal tibia fracture. The patient underwent suprapatellar intramedullary nailing and subsequently developed knee sepsis.

**Conclusion:**

This patient was managed with irrigation and debridements of the knee, fracture site, and intramedullary canal. A resultant soft-tissue defect over the fracture site obviated primary closure. Creation of an acute deformity stabilized by a Taylor spatial frame allowed primary wound closure. After soft tissue healing occurred, the frame was used to correct the intentional deformity and maintain reduction until full healing occurred.

## 1. Introduction

Open tibia fractures are relatively common injuries [1, 2] that can be challenging to treat [3, 4]. Stabilization of these fractures is often performed with intramedullary nailing (IMN), a technique that involves implant placement through either a suprapatellar or infrapatellar portal. Comparison of the two approaches demonstrates that suprapatellar nailing (SPN) facilitates improved alignment, particularly in distal or proximal fractures, [5, 6] and less fluoroscopic usage [7, 8]; however, a potential drawback to SPN of open fractures is iatrogenic knee sepsis as the technique requires intra-articular passage of instruments and the final implant. A 2017 study suggested that knee sepsis following suprapatellar nailing of open fractures does not occur [6], but a 2018 retrospective analysis reported two cases, suggesting a small but present risk for joint contamination [9].

We report the case of a 31-year-old male involved in a dirt bike accident who sustained a type II open tibia fracture and developed knee sepsis following irrigation and debridement (I&D) and suprapatellar nailing. To the best of our knowledge, this is the third documented case of knee sepsis following suprapatellar nailing of an open tibia fracture. Also unique to this case, the patient was definitively treated with a Taylor spatial frame to treat the fracture and also a resultant anteromedial soft-tissue defect at the fracture site following debridement; the frame enabled an intentional deformity to permit primary soft-tissue closure followed by deformity correction. This patient was informed that data pertaining to the case would be submitted for publication, and he provided consent.

## 2. Case Presentation

**Figure 1:**
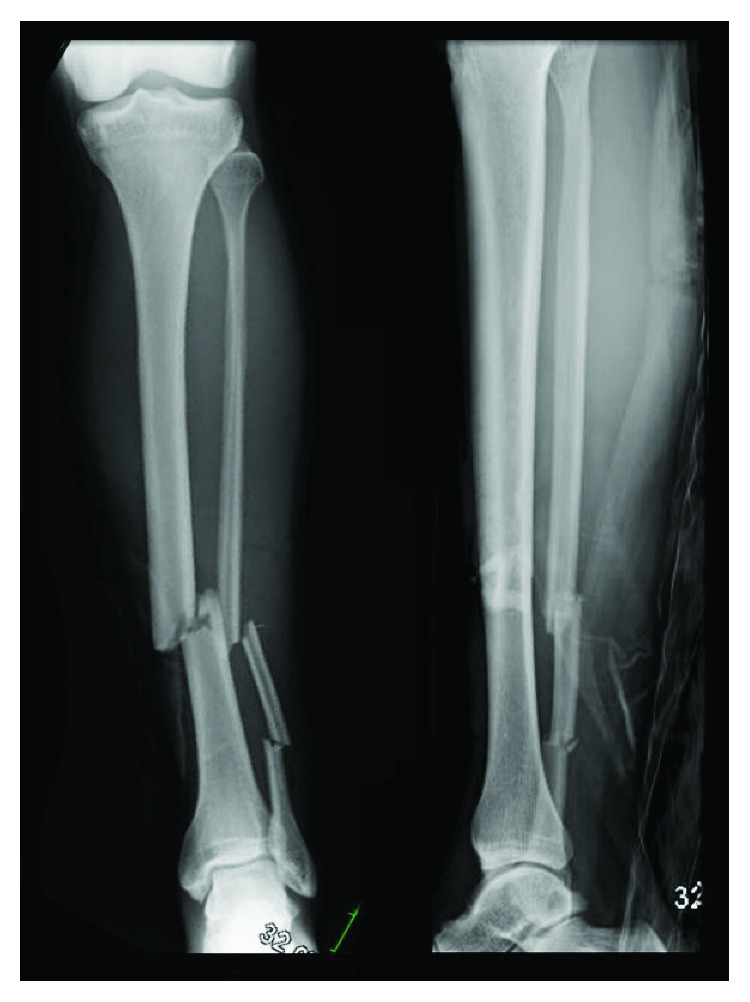
Anteroposterior (AP) and lateral radiographs of the left leg demonstrating a transverse distal diaphyseal tibial fracture and segmental fibular fracture.

An otherwise healthy, nonsmoking, 31-year-old male (25.0 kg/m^2^) was involved in a dirt bike accident and sustained an isolated type II distal diaphyseal tibia fracture with an associated segmental fibula fracture (Figure 1). Neurovascular assessment demonstrated no deficits, and inspection of the leg revealed a 1 cm transverse wound contaminated with grass. The patient initially underwent bedside irrigation and received intravenous (IV) cefazolin, gentamicin, and penicillin G. A splint was applied. Intraoperative irrigation and debridement (I&D) followed by placement of a suprapatellar IMN was completed within the first 24 hours after the patient's arrival. The patient received two doses of IV Ancef postoperatively and was uneventfully discharged with nonweightbearing status on the affected extremity. The patient developed progressive knee discomfort and by week four reported severe pain; examination at that juncture identified a large knee effusion, erythema, and minimal tolerance for knee range-of-motion (ROM). Inspection of the fracture site showed mild erythema with serosanguinous drainage. Aspiration of the knee demonstrated cloudy yellow fluid and, its analysis, a white cell count >100,000 with 96% neutrophils; cultures grew gram-negative rods. At this time, patient care was transferred to the senior author.

I&D of the knee, fracture site, and intramedullary canal was completed following the removal of hardware. Discolored fluid was noted in the knee with palpable loculations in the suprapatellar pouch. Grass was identified in the knee and at the fracture site. A synovectomy of the loculated tissue in the knee was completed, and the surgical footprint at the fracture site was extended proximally and distally to permit local debridement of the bone and soft tissue; nonviable skin was excised resulting in a 1 × 3 cm soft-tissue defect directly over anteromedial tibial fracture site. A reamed irrigator aspirator (RIA) was used to debride the entirety of the intramedullary canal. A total of two separate debridements were performed. Following the second debridement, the soft-tissue defect was too large for primary closure and discussion with the patient included a flap for soft-tissue coverage or use of a Taylor spatial frame with induced deformity to allow primary soft-tissue closure. The patient opted for a frame.

**Figure 2:**
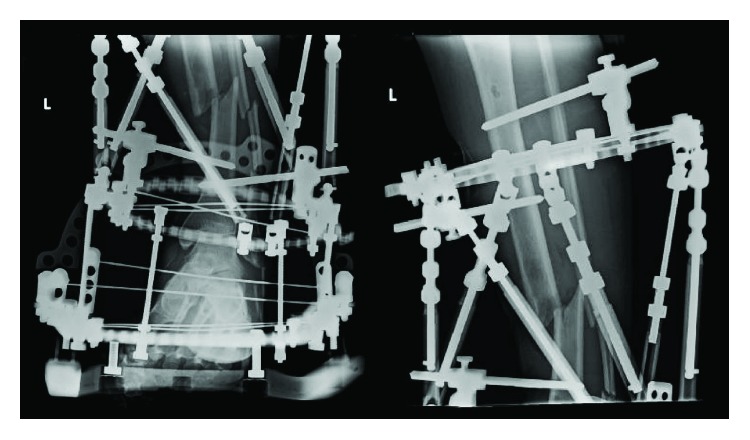
AP and lateral radiographs of the left leg illustrating the induced deformity created with the Taylor spatial frame to allow for primary soft-tissue wound closure.

**Figure 3:**
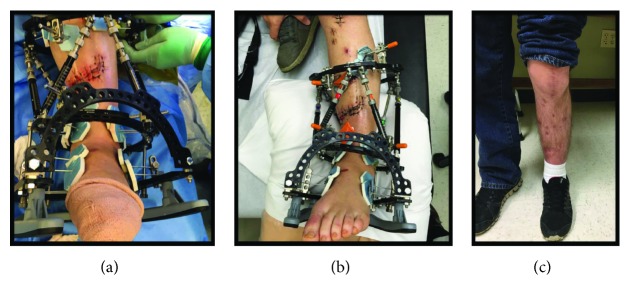
Clinical images demonstrating the acute deformity and soft-tissue closure (a) intraoperatively, (b) 1-week postoperatively, and (c) at final follow-up.

A three-ring frame was applied to the tibia followed by application of Fast-Fx (Smith & Nephew, Memphis, TN) struts between the rings above and below the fracture site. An anatomic reduction of the tibia was completed prior to wound closure, and the strut lengths were recorded; baseline pulses were monitored with a Doppler, and capillary refill was noted. The fracture site was shortened and varus recurvatum deformity was induced until tension-free primary closure of the wound was possible; the struts were fastened, vascular status reassessed, and wound closed (Figures 2 and 3(a)). Postoperatively, the patient had no change in neurovascular status.

**Figure 4:**
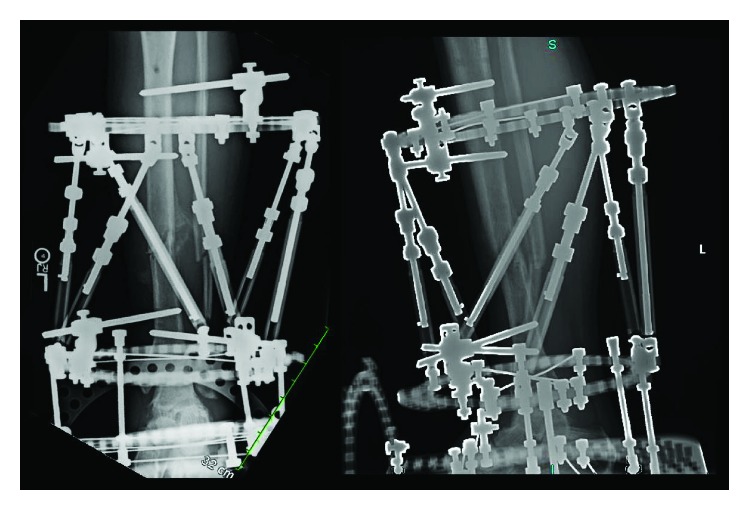
AP and lateral radiographs of the left leg demonstrating deformity correction after a 27-day period.

**Figure 5:**
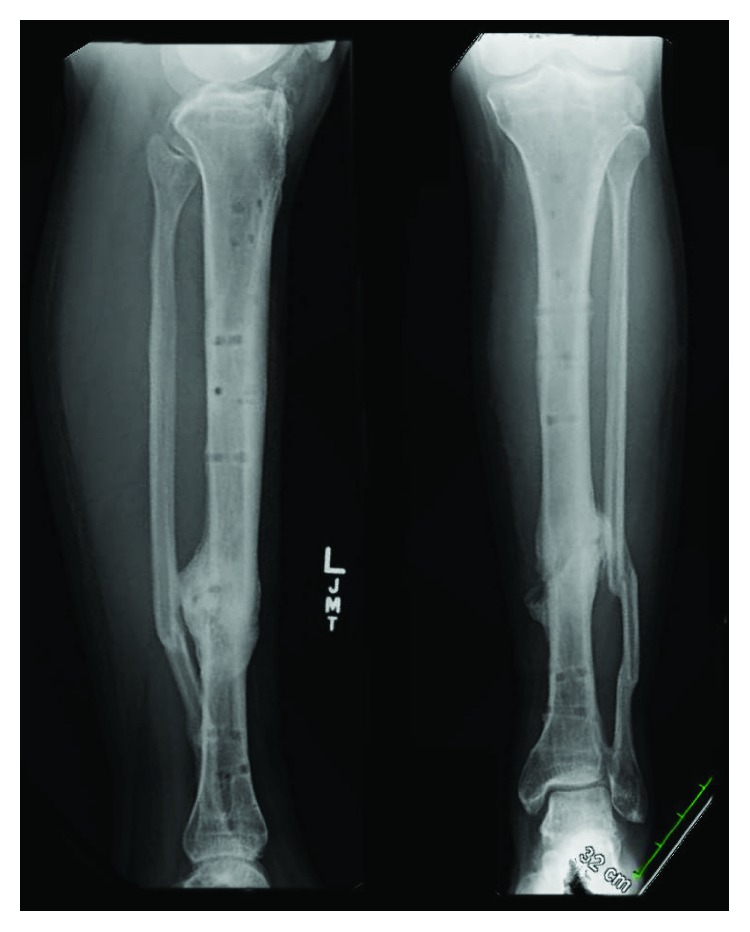
AP and lateral radiographs of the left leg show a united fracture at week 24.

Patient's sutures were maintained for four weeks (wound healed) at which point deformity correction commenced at a rate of 0.75 mm per day for a 27-day period (Figure 4). Throughout the 27-day period, the patient was evaluated weekly, the soft tissues remained intact, near-anatomic alignment was achieved, there were no signs or symptoms of infection, and the patient completed parenteral antibiotics prescribed by the infectious disease team to treat Enterobacter cloacae and Enterococcus faecalis. Once anatomic alignment was achieved, the fracture site was compressed, the foot ring was removed, the patient initiated weightbearing, and the patient was evaluated monthly. At week 24, the frame was removed as radiographic fracture union was achieved (Figure 5). At final follow-up (postoperative month 16), the patient displayed a nonantalgic gait, fully healed surgical sites (Figure 3(c)), and no signs of infection and was working full-time as an auto mechanic.

## 3. Discussion

This report demonstrates a confirmed case of septic arthritis following SPN of an open tibia fracture. Additionally, the patient's soft-tissue defect was closed primarily without a tissue transfer, and the fracture united without internal implants. External fixation following infection and soft tissue loss requires a significant compliance, but this report demonstrates that a desirable result may be achieved. Recently, definitive fixation via suprapatellar IMN has gained favor [5, 6, 9] due to stationary limb positioning resulting in improved fracture alignment and reduced fluoroscopy time [7, 8]. However, the risk of knee sepsis associated with an intra-articular starting point has not been defined in the setting of contaminated open fractures.

In 2017 and 2018, two studies evaluated the rates of knee sepsis associated with suprapatellar nailing of open tibia fractures [6, 9]. Mitchell et al. evaluated 139 open tibia fractures (type II (41%); type III (46%)) treated with suprapatellar nailing and only identified one case of knee sepsis which was attributed to an ascending necrotizing soft tissue infection that ultimately involved the knee joint [6]. Marecek et al. evaluated 289 fractures (type II (31%); type III (56%)) treated with either suprapatellar nailing (*n* = 147) or infrapatellar nailing (*n* = 142). The authors reported two cases (1.4%) of knee sepsis in the suprapatellar group and zero cases (0%) in the infrapatellar group. It was concluded that the risk of knee sepsis following suprapatellar nailing is small but present [9]. Notably, both studies defined knee sepsis by positive aspiration. Marecek et al. reported two patients with “superficial infections” who presented with knee pain, swelling, and erythema. They were treated with antibiotics for presumed septic arthritis but were not included in the knee sepsis category because an aspiration was not obtained. As such, the incidence of knee sepsis following suprapatellar nailing of open fractures may be underestimated.

A common treatment for open tibia fractures with significant soft-tissue defects is the “fix and flap,” which utilizes internal fixation and a rotational or free flap to close the soft-tissue defect [10, 11]. In our patient, there was a concern that placing an internal implant in the setting of knee sepsis contaminated intramedullary space, and soft tissue infection might create another substrate for microorganism colonization. While there is evidence supporting the maintenance of internal hardware in the setting of an acute postoperative infection [12], predictors of failure included the presence of an IMN or gram-negative infection, variables that were both present in our patient. Others suggest that external fixation for severe open tibial fractures may reduce the risk of infections when compared to internal fixation because no hardware is placed at the fracture site [13, 14]. In our patient, ring fixation ultimately permitted fracture stabilization in the setting of a postoperative infection and also enabled a concomitant soft-tissue reconstruction.

The use of intentional deformity to facilitate soft-tissue reconstruction has been described for a variety of posttraumatic conditions [15–17] including its combined use with local rotational flaps for large soft-tissue defects of the leg [18]. While the use of either a rotational flap or free-tissue transfer remains the standard of care, the factors that may limit its use include lack of access to a microvascular surgeon, patient preference, or patient is not a candidate for such a soft-tissue procedure. The current report further highlights that acute shortening with angular deformity can be effective and avoid the need for flap. In our case, the factors that permitted the technique to work included a soft-tissue void that could be primarily closed without compromising vascularity to the foot and having a fibula fracture at a level near that of the tibial injury to allow deformity in the direction of soft tissue loss.

Finally, given the intraoperative findings in our case, it is important to highlight the importance of meticulous debridement with wide excision of the involved soft tissue and bone in the surgical management of any open fracture.
